# Three Shifts That Will Redefine Health Systems

**DOI:** 10.1002/hcs2.70051

**Published:** 2026-02-15

**Authors:** You Wu, Haibo Wang, Zongjiu Zhang

**Affiliations:** ^1^ School of Healthcare Management, Tsinghua Medicine Tsinghua University Beijing China; ^2^ Institute for Hospital Management Tsinghua University Beijing China; ^3^ Research Centre of Big Data and Artificial Intelligence for Medicine, The First Affiliated Hospital Sun Yat‐sen University Guangzhou China

Academic journals, consulting firms, and mainstream media have often published “predictions” about the future development of medical and health care. These publications often emphasize the potential of cutting‐edge scientific or technical breakthroughs. *Health Care Science* looks at the problem from another perspective. We focus on how these changes enter the health system, how they operate in the real world, and how they reshape the organization and governance of medical services. At the beginning of 2026, we envision the following three major shifts that will reshape healthcare.

## 1 From Treatment to Prediction and Prevention

Although the concept of shifting “from sick care to health care” has been discussed for more than a decade [1], healthcare systems are still largely organized around treating disease after it appears. Patients develop symptom, seek care, and health providers respond. This approach is gradually changing, as advances in data collection, monitoring, and risk modeling allow earlier identification of health risks and diseases, sometimes even before clinical symptoms emerge.

Effective prevention also depends on the built healthy environment of the population. Our living conditions, nutritional status, and physical capacity are the foundations, based on which disease prevention can be translated into health benefits. This change will also require adjustments in health financing models, and shift the window of performance evaluation to cover health benefits such as DALY or QALY saved.

For health services research, we propose the following research priority: when should predicted risks lead to intervention; how to avoid overdiagnosis and unnecessary anxiety among patients; how to coordinate across hospitals, public health sectors, community and society; and how to ensure that preventive strategies benefit all populations rather than widening inequalities [2].

## 2 Artificial Intelligence (AI)‐Enabled Digital Transformation in Healthcare

Patients today expect timely, seamless, and accessible healthcare services across everyday settings—at home, in workplace, even during travels. Medical services should strive to meet the needs of patients and rethink how care is organized and delivered. Wearable devices and sensors have expanded the scope of health‐related data, making it possible to gather information that was previously difficult to obtain outside traditional healthcare settings [3]. Meanwhile, AI is no longer limited to completing single task; it is streamlining daily clinical operations. Technology could be the solution, but its impact will depend on how well it is integrated into the existing health systems. That is why we see digital health platforms becoming critical infrastructures to connect hospitals, providers, and patients across different stages of care.

As these technologies move from development into deployment, governance frameworks became a central challenge [4]. Unintended consequences have been reported from early AI uses in academic and clinical settings [5, 6]. In response, the World Health Organization has advocated for, and many countries have begun to implement, lifecycle–based regulatory frameworks especially for AI as a Medical Device (AIaMD) [7]. In addition to health services delivery, AI and digital tools are also changing how evidence is generated from health system [8]. From bench to bed and then back to bench, AI is accelerating the convergence of biological and clinical research [9]. Although amid all hypes and hopes, recent evidence has raised concerns about a “collective narrowing of scientific focus” as AI becomes more deeply embedded in academia [10].

We believe that AI‐enabled health systems require clear rules of accountability, transparency, safety and human oversight. Patients' trust in these systems will depend on formal evaluation frameworks, rigorous research design that can keep up with fast‐evolving algorithms, and the ability to identify and correct unexpected consequences [11]. In our first editorial in 2022, *Health Care Science* stated the aim to “bring the world new perspectives on medical innovations and healthcare systems, through the lenses of legislation, policy, ethics, and humanity”. That mission feels more urgent than ever today.

## 3 From Patient‐Centric Care to Patient Engagement

The third major shift involves the role of patients in health care. Although “patient‐centered” care has always been a stated goal, nowadays our priority is delivering value‐based care [12], that is, care should be not just *around* the patients, but genuinely *for* the patients,. Emerging technologies are enabling patients to participate in the whole process from prevention, diagnosis to treatment, rehabilitation and long‐term management, defining what they consider as “valued.” They will gradually gain the capacity to interpret medical information that was once exclusive to professionals. These abilities allow patients greater autonomy in choosing treatment options and medical service providers according to their personal needs and preferences.

This change will reshape clinical encounters, informed consent process, treatment compliance and accountability, bringing new opportunities to the medical system, meanwhile creating tensions as well [13]. At the same time, we call for awareness that not all patients have equal resources to engage, therefore health systems must ensure that we enhance, rather than undermine, fairness and trust [14].

## 4 Looking Ahead

These three shifts are closely interconnected. Predictive and preventive care reshapes the goals of healthcare. AI and digital infrastructures transform how care is delivered. Patient engagement affects how care is experienced and evaluated. Together, we expect health systems to become more integrated and participatory. As 2026 begins, the editorial team of *Health Care Science* is looking forward to more research that examines these trends with methodological rigor and practical relevance.

To echo what our editors‐in‐chief wrote in the inaugural issue [15], “The breakthroughs in medical technology and clinical research are exciting but never the panacea.” What *Health Care Science* cares about is the trials and errors that bring these solutions to reality.

## Author Contributions

**You Wu:** writing – original draft, investigation. **Haibo Wang:** writing – original draft, writing – review and editing, investigation. **Zongjiu Zhang:** investigation, writing – original draft, resources, supervision.

## Funding

The authors have nothing to report.

## Ethics Statement

The authors have nothing to report.

## Consent

The authors have nothing to report.

## Conflicts of Interest

Professor Zongjiu, Zhang, Haibo Wang, and You Wu are members of the *Health Care Science* Editorial Board. To minimize bias, they were excluded from all editorial decisionmaking related to the acceptance of this article for publication.

## Data Availability

The authors have nothing to report.
